# Welfare-aligned Sentience: Enhanced Capacities to Experience, Interact, Anticipate, Choose and Survive

**DOI:** 10.3390/ani9070440

**Published:** 2019-07-13

**Authors:** David J. Mellor

**Affiliations:** Animal Welfare Science and Bioethics Centre, School of Veterinary Science, Massey University, Palmerston North 4442, New Zealand; d.j.mellor@massey.ac.nz; Tel.: +64-21-390-855

**Keywords:** affective neuroscience, conscious subjective experiences, critical anthropomorphism, indicative behaviours, invertebrates, sensory inputs, vertebrates

## Abstract

**Simple Summary:**

Formal recognition that some animals are sentient beings is now widespread and continues to increase internationally. Sentience is a capacity of animals to consciously perceive by the senses; to consciously feel or experience subjectively. In animals that manifest different states of welfare, these experiences can be negative, that is, potentially welfare compromising, or positive, that is, potentially welfare enhancing. As there have been significant advances during the last two decades in the science that underpins our understanding of sentience, the major purpose here is to provide up-to-date perspectives on that understanding. Thus, the present focus is on the key features of sentience in animals which can experience different states of welfare, encapsulated by the new term ‘welfare-aligned sentience’. This term is intended to exclude potential forms of sentience that do not enable animals in some taxa to have the subjective experiences which underlie different welfare states. The approach adopted is to present 11 interconnected statements about sentience-associated body functions and behaviour, and to explain them in largely non-technical language. Topics covered include the following: The characteristics of nervous systems required for welfare-aligned sentience to be expressed and how those characteristics develop in young animals; the importance of sensory inputs from inside the body and from outside the body and their roles in generating particular sensations, feelings, emotions and other subjective experiences; how these experiences elicit behaviours that help the animal to survive, and are also key elements in animals’ communication with others and their interactions with the environment. The following are also considered: How this new scientific knowledge helps to circumvent some acknowledged pitfalls of anthropomorphism when making inferences about the particular subjective experiences that animals may have; and the possible inclusion of more invertebrates among the list of animals that possess a capacity for sentience—a list which, to date, has been dominated by vertebrates. In addition, it is noted that the earlier assessment of the presence or absence of sentience by predominantly exploring responses to potentially aversive experiences such as pain, needs to be revised. Such assessments should also include sentient animals’ capacities to have and behaviourally express positive subjective experiences. Finally, the following succinct definition is offered for consideration: Welfare-aligned sentience confers a capacity to consciously perceive negative and/or positive sensations, feelings, emotions or other subjective experiences which matter to the animal.

**Abstract:**

The focus of this opinion is on the key features of sentience in animals which can experience different states of welfare, encapsulated by the new term ‘welfare-aligned sentience’. This term is intended to exclude potential forms of sentience that do not enable animals in some taxa to have the subjective experiences which underlie different welfare states. As the scientific understanding of key features of sentience has increased markedly during the last 10 to 15 years, a major purpose here is to provide up-to-date information regarding those features. Eleven interconnected statements about sentience-associated body functions and behaviour are therefore presented and explained briefly. These statements are sequenced to provide progressively more information about key scientifically-supported attributes of welfare-aligned sentience, leading, in their entirety, to a more comprehensive understanding of those attributes. They are as follows: (1) Internal structure–function interactions and integration are the foundations of sentience; (2) animals posess a capacity to respond behaviourally to a range of sensory inputs; (3) the more sophisticated nervous systems can generate subjective experiences, that is, affects; (4) sentience means that animals perceive or experience different affects consciously; (5) within a species, the stage of neurobiological development is significant; (6) during development the onset of cortically-based consciousness is accompanied by cognitively-enhanced capacities to respond behaviourally to unpredictable postnatal environments; (7) sentience includes capacities to communicate with others and to interact with the environment; (8) sentience incorporates experiences of negative and positive affects; (9) negative and positive affective experiences ‘matter’ to animals for various reasons; (10) acknowledged obstacles inherent in anthropomorphism are largely circumvented by new scientific knowledge, but caution is still required; and (11) there is increasing evidence for sentience among a wider range of invertebrates. The science-based explanations of these statements provide the foundation for a brief definition of ‘welfare-aligned sentience’, which is offered for consideration. Finally, it is recommended that when assessing key features of sentience the same emphasis should be given to positive and negative affective experiences in the context of their roles in, or potential impacts on, animal welfare.

## 1. Introduction

The rising interest during the last 30–40 years in the notion, or reality, that animals of particular taxa possess a capacity for sentience may simply be the most recent oscillation in the balance of thinking between skepticism and confidence on this matter expressed by philosophers, scientists and other professionals over the last 300 years [1]. At the same time, it seems likely that many, possibly most, lay people believe, and have long believed, that animals are sentient, a belief based on common sense. Several factors have contributed to the recent surge in professional confidence that animals of various taxa do indeed exhibit at least some hallmarks of sentience, which includes having sensations, feelings, emotions or other subjective experiences, generically known as affects. These factors include the inception of animal welfare science, and within that burgeoning discipline, the emergence of new evidence and the consolidation of the ‘affective state’ orientation as a key element in understanding the welfare of animals (e.g., [1,2,3,4,5,6,7,8,9,10,11,12]).

Indeed, this surge in confidence has led to increasingly widespread international declarations that animals whose welfare is of concern are sentient beings [13], for example, within the European Union via the Treaty of Lisbon (2008); via laws in France (2015), New Zealand (2015) and Quebec (2015); by at least 46 countries which supported a proposal that the United Nations issue a Universal Declaration on Animal Welfare [14]; and by the 180 member countries of the World Organisation for Animal Health (OIE), which, in adopting the OIE Global Animal Welfare Strategy 2017, accepted a statement recognising animal sentience [15]. 

This formal science-based recognition that animals such as vertebrates are sentient explicitly counters what was disallowed by the earlier behavioural science dogma that dominated thinking during the 70 years or so before about 1995, that is, that animals’ behaviour could be described, but that anthropomorphic obstacles meant it was not scientifically meaningful to interpret behaviour in terms of the animals’ motivations and/or what they may be experiencing subjectively (see Proctor [16]). This view was challenged strongly by Jaak Panksepp [17,18,19,20], who, together with numerous others, provided compelling neurophysiological, affective neuroscience, veterinary clinical science and behavioural science evidence that supported the existence and welfare significance of key attributes of sentience among those animals that exhibit them through their behaviour (e.g., [5,7,11,21,22,23,24,25,26,27,28,29,30,31,32,33,34,35,36,37,38,39,40,41,42,43,44,45,46,47,48,49]). Although vertebrates are the primary focus of this opinion, it is acknowledged that evidence is accumulating of unexpectedly impressive cognitive capacities and behavioural flexibility associated with subjective experiences in a number of invertebrates, which mostly have much simpler nervous systems [50].

The major purpose here is to provide up-to-date information on the science that underpins understanding of the way sentience is expressed in those animal taxa which can experience different states of welfare, encapsulated by the new term ‘welfare-aligned sentience’. This term is intended to exclude potential forms of sentience that do not enable animals in some taxa to have the subjective experiences which underlie different welfare states. To this end, 11 interconnected statements about sentience-associated body functions and behaviour are presented and briefly explained in largely non-technical language. An extended abstract of an earlier, much shorter version of this paper has been published by the The Royal Society for the Prevention of Cruelty to Animals (RSPCA) UK [51]. The reader is also referred to a 2017 discussion by diverse sectoral representatives, industry groups and other organisations regarding the implications of giving statutory recognition to animal sentience in the 2015 amendment of New Zealand’s Animal Welfare Act 1999 [52].

## 2. General Statements about Welfare-Aligned Sentience

This sequence of statements is designed to provide progressively more detail regarding key scientifically-supported attributes of welfare-aligned sentience, leading, in their entirety, to a more comprehensive understanding of those attributes. The statements should therefore be considered as a whole, not each one in isolation.

### 2.1. Internal Structure–Function Interactions and Integration are the Foundations of Sentience

Each sentient animal is a living embodiment of dynamically unified and integrated forms, functions, behaviours and related capacities to have subjective experiences, features from all of which are observable in combinations that are unique to each species, evolved to secure survival within particular environments (for references see [53,54]). Each such animal therefore expresses discoverable forms of biological coherence whilst operating as a whole entity within its ecological niche (e.g., [53]).

### 2.2. Animals Exhibit a Capacity to Respond Behaviourally to a Range of Sensory Inputs

At a basic neurophysiological level, sensory inputs take the form of electrical impulses generated by specialised nerve endings (receptors) that respond to pressure (touch, vibration, stretch), chemical (smell, taste), thermal (heat, cold), sound (hearing), light (sight) and/or other stimuli. These impulses pass along specific nerves to more complex neural structures that process them in ways that generate impulse outputs in other nerves, some of which function to elicit or modify particular behaviours. The processing neural structures exhibit different degrees of complexity across taxonomic groups. They progress from sophisticated nervous systems that incorporate a spinal cord and brain (e.g., in vertebrates), through large brains connected to extensive nerve networks (e.g., in cephalopods), to chains of clustered nerve cell bodies (ganglia) (e.g., in crustaceans and insects), and basic nerve cord networks (e.g., in earthworms), to even simpler forms [55,56].

### 2.3. The More Sophisticated Nervous Systems can Generate Subjective Experiences (i.e., Affects)

The processing of sensory inputs by the nervous systems of vertebrates, for example, can generate behaviourally-relevant subjective experiences. These affective experiences include sensations, feelings and emotions [2,4,7,11,35,38,45,57,58], many, if not most of which contribute to eliciting, or accompany, particular behaviours (see Statements 2.7 and 2.9). Importantly, the major features of brain processing of such sensory inputs appear to be common to all higher animals, including mammals, birds and fishes [19,39,42,59,60,61,62].

### 2.4. Sentience Means that Animals Perceive or Experience Different Affects Consciously

Sentience is defined as having a capacity to *perceive* by the senses; a capacity to *feel* or *experience* subjectively [63]. For something to be *perceived* or *experienced* the animal must be conscious. Thus, the words *perceive* and *experience* are taken here to mean to *consciously* perceive and to *consciously* experience. This means that the animal is aware of sensations, feelings, emotions and other subjective outputs of the neural processing of sensory inputs generated both from within the body and outside it [64,65]. By this definition, therefore, animals that are sentient must possess a capacity to be conscious [63]. Accordingly, not only does sentience require the operation of nervous systems of sufficient sophistication to detect and process various sensory inputs in ways that give rise to a range of subjective (affective) experiences (for references see [66]), these nervous systems, whatever their level of complexity, must also have a capacity to express, and/or sustain a state or states of consciousness.

An animal may be inferred to be conscious when it exhibits behavioural flexibility, including capacities to direct attention towards relevant stimuli, to determine situation-appropriate responses under novel conditions and to engage in volitional, goal-directed behaviours [49,67,68].

### 2.5. Within a Species, the Stage of Neurobiological Development is Significant

In species currently regarded as sentient (e.g., vertebrates), there is a period after conception when the developing nervous system is too immature to support consciousness, which, as already noted is a key attribute of sentience (Statement 2.4). There follows an intermediate phase when it is unclear whether or not some basic forms of consciousness and, therefore, sentience might be possible; the implications of this remain to be determined [69], but see Statement 2.10. In the young of land mammals, for example, this phase ends when neural connections become established between the cerebral cortex and the lower regions of the brain (for references see [66]). These connections enable cortically-based forms of consciousness to support the experiential capacities of these mammals from the time they appear after birth and, thereafter, potentially for the rest of their lives. These forms of consciousness become apparent several months after birth (e.g., in marsupials), several days-to-weeks after birth (e.g., in bears, cats, dogs, ferrets, hamsters, mice, rats and rabbits), or several minutes-to-hours after birth (e.g., in guinea-pigs, ungulates, such as horses, cattle, goats, sheep, pigs, and many primates) (for references see [66,70,71,72,73]). Thus, achievement of this cortically-based sentience milestone [71,73] occurs just before the time when these young first leave the maternal pouch or the protective den, nest or other enclosure, or when, straight after birth outdoors, the young first enter a relatively unprotected external environment [66]. Interestingly, the chicks of different avian species also possess a wide range of neurological maturity and sensory capacities at hatching, and, paralleling parent–offspring interactions in mammals, these varied neurological capacities influence post-hatching parent-focused chick behaviour and, reciprocally, the character and duration of chick-focused parental behaviour [74,75,76].

### 2.6. During Development the Onset of Cortically-Based Consciousness is Accompanied by Cognitively-Enhanced Capacities to Respond Behaviourally to Unpredictable Postnatal Environments

The onset of cognitive awareness confers a high degree of behavioural flexibility that allows the young to respond more effectively to the unpredictability of the environments they encounter after birth [66]. In line with Statement 2.5, this cortically-based, *enhanced cognitive capacity* takes several months, days-to-weeks or minutes-to-hours before it is expressed behaviourally in these different groups of young mammals. It is apparent that the onset of this cognitively-enhanced flexibility in the young of each group coincides with their first exposure to variable environments that require such volitional behavioural responsiveness [66]. This has timely survival-enhancing implications, which continue for the rest of the animal’s life [66].

### 2.7. Sentience Includes Capacities to Communicate with Others and to Interact with the Environment

The ability of higher order animals such as vertebrates to communicate or otherwise interact within their own species and with other species, which sometimes includes human beings, is an indication of their sentience. Communication involving transfers of information between senders and receivers requires engagement of their externally directed sense organs, for example, for touch, temperature, taste, smell, hearing and/or sight, and interpretation of the associated signals [54,77,78]. Communication may be intra-specific or inter-specific, active or passive, benign or threatening, routine or novel, and may have numerous purposes and/or consequences that often involve affective experiences for both senders and receivers [77].

One or more of the six most familiar senses may exhibit an exaggerated capacity [79]; this enables the affected species to successfully engage with what would otherwise be insurmountable challenges posed by their ecological niche [54,66]. Examples include the exaggerated acuity of sight in eagles [80], smell and ultrasonic hearing in dogs [81,82,83,84,85,86], and sight, smell and ultrasonic hearing in cats [85,87]. In addition to enhancing the scope of effective behavioural responses in particular environments, such exaggerated sensory capacities likely also influence the modes of communication utilised by these animals. Other evolved capacities include the rare sensory modality of ultrasonic echolocation that aids toothed whales, dolphins, some bats, and swifts to find their way in low light-intensity environments [88], the specialised receptors possessed by sharks and rays that enable them to detect weak electromagnetic fields generated by living prey [89,90], mechanoreceptors in the lateral line organs of some fish that enable them to detect the movement of other conspecifics during non-contact territorial interactions [91], and the unusual chemical sensitivity of the forked tongue in reptiles that confers on them heightened abilities to identify prey, recognize kin, choose mates, locate shelters and follow trails [92,93]. 

### 2.8. Sentience Incorporates Experiences of Negative and Positive Affects

Brain function in many vertebrates enables the expression of sentience to include a capacity to consciously experience and distinguish between negative and positive affective experiences. This capacity to recognise the valence of different affects has direct relevance to the animal’s welfare. This is because negative affects, which have intensities and/or durations above tolerably low levels, tend to be welfare compromising, whereas positive affects tend to be welfare enhancing [11,46,47,49,68,94,95]. Negative and positive affective experiences, therefore, have animal welfare significance, so both must be considered. Sentience, as possessed by such animals, therefore incorporates a capacity to subjectively experience negative and positive sensations, feelings or perceptions that matter to the animal because they affect its welfare [9,53,54,96].

### 2.9. Negative and Positive Affective Experiences ‘Matter’ to Animals for Various Reasons

The word ‘matter’ is well chosen. In animal welfare terms it relates mainly to negative and positive experiences [9], but usually not to operationally-neutral affects [45]. Examples of neutral affects are the subjective experiences associated with the functionality of vision, hearing, vocalisation and proprioceptive sensing of posture, position or motion, all of which remain neutral unless the operation of these senses is compromised in some way.

#### 2.9.1. Negative Affective Experiences

There are two main types of negative experiences, that is, *survival critical* ones, which motivate animals to engage in particular behaviours; and *situation-related* ones, which reflect the animal’s perception of its external circumstances [45,53,95]. 

The *survival-critical negative experiences*, which are usually generated by disturbances or imbalances in physiological states within the animals, matter in a positive way. That is because, in being negative, they impel the animal, that is, motivate it with a sense of urgency to engage in behaviours aimed at correcting the associated internal disturbances or imbalances, thereby helping to secure the animal’s survival. Examples include breathlessness to restore normal oxygen supply when breathing is compromised, thirst to elicit drinking to correct dehydration, hunger to motivate eating to restore energy supply, and pain for escape from or to avoid injury [11,45,53,95]. These experiences also matter in a negative way. This is because when animals are unable to successfully engage in the motivated behaviours, the disturbances or imbalances are not corrected and may get worse, so that the associated negative experiences persist and their unpleasant, aversive or noxious intensity often increases markedly.

The *situation-related negative experiences* mainly reflect the animal’s perception of its external circumstances and include, for example, frustration, anger, helplessness, loneliness, boredom, depression, anxiety, fear, panic and nervous vigilance (see: [11,19,22,23,29,34,35,49,53,95,97,98,99,100]). Examples of conditions likely to elicit these experiences are as follows: Invariant, barren features of indoor or outdoor enclosures; very limited space; severely restricted opportunities to engage in environment-focused exploration and/or interactive social behaviours; and/or an inability to escape from being threatened by others when kept in groups [19,34,35,45,46,47,49,97,99].

#### 2.9.2. Positive Affective Experiences

*Positive experiences* ‘matter’ in two ways. First, because the activities or situations that give rise to them may variously be engaging, stimulating, enlivening, emotionally rewarding, physically satisfying and/or otherwise enjoyable (e.g., [37,45,46,47,99,101]). Second, because when an animal anticipates them, participates in generating them and then recalls them after the event, positive feelings, emotions or subjective perceptions enhance the overall hedonic experiences that animals may have in ways that may improve their quality of life (e.g., [7,9,11,12,45,46,95,102,103,104,105,106]). In general terms, the associated positive affective experiences are considered likely to include various forms of comfort, pleasure, interest, confidence and a sense of being in control (for references see [7,11,37,99,107]). 

### 2.10. Acknowledged Obstacles Inherent in Anthropomorphism are Largely Circumvented by New Scientific Knowledge, but Caution is Still Required 

A problem highlighted by animal behaviour scientists last century (see [1,19]) and by other scientists more recently (e.g., [9,36,108]) is that interpretative complications, due to anthropomorphic projection, may arise when affective experiences in animals are being considered (also see: [16,109]). During the last 20–25 years, however, knowledge of the neurophysiological mechanisms that underlie the generation of particular affective experiences has markedly increased (e.g., [5,7,11,17,18,19,20,21,22,23,24,25,26,27,28,29,30,31,32,33,34,35,36,37,38,39,40,41,42,43,44,45,46,47,48,110]), so that cautious application of this extensive knowledge now largely circumvents general concerns about anthropomorphism [16,46,47,95]. It follows that the more extensive this knowledge is in a particular species, the greater the confidence level assigned to conclusions about the range of different types of affects experienced by that species can be. In contrast, when little such knowledge is available, the confidence level assigned to these conclusions will be proportionately lower and the caution required when formulating them proportionately higher [68,111,112]. For example, detailed knowledge of the extent and character of affect-related behaviour in many wildlife species, including terrestrial and aquatic mammals and birds, is often very limited, as it is in reptiles, amphibians and fishes (e.g., [68,78,107,113,114,115,116,117,118,119,120,121,122,123]).

Another more specific objection related to the claimed pitfalls of anthropomorphism may now be seen to reflect a conceptual misconstruction. It is true that the scientific focus on particular affects in animals is inevitably and crucially influenced by human experience [16]. Accordingly, without such direct experience the subjective character of alien sensory capacities (Statement 2.7), for example, ultrasonic echolocation, remains a mystery. However, humans and other animals, especially many terrestrial mammals and probably birds, are considered to share in common at least some of the specific experiences of *survival-critical affects* such as breathlessness, thirst, hunger, pain, nausea, dizziness, debility, weakness and sickness (e.g., [4,5,11,19,38,44,45,58,95,105,124]). This view is strengthened because these affects are generated by sensory inputs that register specific functional disturbances or imbalances within the body which, when they are extreme, would threaten the survival of most mammals and birds [11,45]. Recall that the function of each of these negative affective experiences is to motivate behaviours that are designed to correct the particular internal disturbance or imbalance that originally generated the affect (see Statement 2.9). Also note that the presence of such functional disturbances or imbalances may be confirmed by definitive physiological, pathological, clinical and other such evidence in the mammals and birds of interest [11,45].

The misconstruction therefore arises when critics interpret the above view as meaning that the subjective character of each of these affective experiences is the same, that is, identical in humans and in other named animals. This interpretation is inaccurate. Rather, a more informative interpretation is that the motivational significance of the negative character of each affect for the animal *in the animal’s terms* is similar to the motivational significance of the same affect for a human *in human terms*. In other words, the focus is not on the identicality of the precise character of the affective experiences in humans and other animals; rather, it is on the equality of its capacity to motivate particular behaviours.

It follows that when there is strong neuroscientific support for the existence of a motivational connectedness between specific *survival-critical affects* and the *corrective behaviours* they elicit in a particular species, careful observation of behaviour alone will usually enable conclusions about the likely presence and roles of specific affects to be drawn with a high degree of confidence [112]. For example, it has been well demonstrated that osmoreceptor activity elicited by dehydration generates the experience of thirst which, in turn, motivates water-dinking that corrects the dehydration, decreases osmoreceptor activity and thereby quenches the thirst [38]. This scientific understanding, therefore, supports a confident conclusion that thirst motivates the urgent water-seeking and drinking by animals which had previously been deprived of water for lengthy periods in hot weather.

*Situation-related negative affects* reflect the animal’s perception of its external circumstances [45,95], and in many terrestrial mammals they are considered to include frustration, anger, helplessness, loneliness, boredom, depression, anxiety, fear, panic and nervous vigilance (e.g., [11,19,22,23,29,34,35,49,97,98,99,100]). However, identifying and distinguishing between some of these affects may be more susceptible to anthropomorphic error than is the case with the survival-critical affects. On the one hand, provided that the particular circumstances of the animal are borne in mind, there are sound neuroscience bases for using indicative behaviours to distinguish between anxiety, fear, panic, depression, frustration and anger with a reasonable level of confidence (e.g., [5,18,19,20,28,29,36,41,42,48]). On the other hand, in view of a significant overlap in some of their indicative behavioural features, such as isolated withdrawal indicated by inactivity and low interactivity, distinguishing between helplessness, loneliness and/or boredom on the basis of behaviour may require much greater caution [22,23,34,48,100,112].

The existence of *situation-related positive affects*, recently characterised as aligned with states of ‘positive affective engagement’ [46], is strongly supported by extensive affective neuroscience observations. In particular, the neuroscience of reward-seeking and the generation of positive affects supports the interpretation that animals will likely have pleasurable experiences when exhibiting behaviours that include the following [47]: positively motivated, energised environmental exploration and food acquisition activities, that is, activities which are not motivated by significant negative survival-critical affects (e.g., thirst, hunger); bonding and bond affirmation; maternal, paternal or group care of young; play behaviour; and sexual activity (e.g., [17,19,21,24,25,26,30,33,35]). These largely neuroscience-supported inferences from animal behaviour generally accord with, and are thereby strengthened by, prior interpretation of predominantly behaviour-based investigations of animal preferences, aversions and priorities conducted independently (e.g., [22,31,32,34,40]). Thus, provided that key features of an animal’s circumstances are well known, the above observations support the confident use of indicative behaviours to identify states described in general terms as ‘positive affective engagement’, which, as noted above, may include various forms of comfort, pleasure, interest, confidence and a sense of being in control (e.g., [7,37,45,99,101,107,125]).

The above approach to evaluating affects incorporates some of the key features of *critical anthropomorphism* [126]. These include utilisation of extensive affective neuroscience and behaviour science knowledge, detailed understanding of the way animals behaved in precisely described circumstances, and, in each case, considering the general level of confidence that can be assigned to what is actually known in order to guide the caution with which inferences about affects can be made. 

### 2.11. There is Increasing Evidence for Sentience among a Wider Range of Invertebrates 

The analysis to this point has primarily focused on vertebrates, which are generally regarded as being sentient. However, several questions remain to be addressed. Which animals are and which are not sentient? Is there a clear dividing line between these two categories? Is there a scale of different levels of sentience? 

It seems to be the case that animals in all taxa examined to date respond behaviourally to external stimuli in ways that are apparently survival orientated, where such responses across taxa range from being very complex or sophisticated to very limited or simple. However, distinguishing between those animals which possess a capacity for sensory inputs to generate conscious subjective experiences that influence their behaviour (i.e., sentience), and those that merely respond automatically to sensory inputs without an awareness of subjective experiences (i.e., insentience), remains problematic. The present analysis side-steps making this distinction by focusing on features of sentience that underlie the capacities of animals in different taxa to have negative and/or positive subjective experiences that are primary determinants of their welfare state, encapsulated by the new term ‘welfare-aligned sentience’.

Nevertheless, it is worth noting that the phylogenetic distinctions made in the past between sentient and insentient species have become increasingly blurred. More is known regarding potential substrates of sentience in vertebrates than in invertebrates [127], in part, because until recently, the possibility that invertebrates could consciously experience emotion-like states had been largely dismissed [50], cephalopods being a notable exception [1,56,127,128,129,130]. However, accumulating literature is now providing evidence of impressive cognitive capacities and behavioural flexibility in some other invertebrates. Thus, crayfish, sea crabs, slugs, snails, bees, flies and ants have all been shown to display various cognitive, behavioural and/or physiological phenomena that suggest the existence of internal states reminiscent of emotions [50]. Moreover, these observations suggest that the possession of a small brain or simple nervous system does not necessarily rule out a capacity to experience emotion-like states that might motivate or at least accompany specific behaviours [50]. However, the information currently available for most invertebrates is insufficient to indicate whether or not their welfare should be raised as a significant area of concern. 

One approach to decisions about which species are and are not sentient is to conservatively include only those animals whose sentience can be asserted with some confidence; another is to extend the compass of concern by adopting the precautionary principle, thereby giving animals on the margin ‘the benefit of the doubt’ with regard to their sentience [131,132]. This has practical relevance when considering which animals should be protected by animal welfare legislation. Interestingly, the New Zealand Animal Welfare Act 1999 effectively adopted both approaches by confidently including all living vertebrates and by cautiously including specific invertebrates, namely any octopus, squid, crab, lobster, or crayfish (including freshwater crayfish). It also mandated extending this list from time to time by enabling the Governor General, via Orders in Council, to specify additional animals to be covered by the Act. In addition, the precautionary principle was applied with regard to inclusion of developmental stages. Thus, sentient animals are considered to also include any mammalian fetus, or any avian or reptilian pre-hatched young, that is in the last half of its period of gestation or development, plus marsupial pouch young. This might appear to be over-cautious if attention is focused on the postnatal onset of cortically-based consciousness or the equivalent after hatching in birds [66,70,71,72]. However, it is argued here that such caution will remain appropriate until the question of whether or not, during the prior neurodevelopmental phase (Statement 2.5), brain function can support restricted forms of welfare-aligned sentience and associated basic forms of consciousness. This may be especially important for those young in which cortically-based consciousness (mammals), or its equivalents (birds), appears several months or days-to-weeks after birth or hatching [69].

## 3. Key Features of ‘Welfare-Aligned Sentience’—A Summary and Definition

Statutory acknowledgements that (some) animals are sentient have usually not included a definition, and this has understandably led diverse groups with an interest in the operational implications of sentience to call for it to be defined (e.g., [51,52,133]). This is no easy matter because a definition must be specific enough to provide a direction for policy development and to be useful legally. On the other hand, a definition needs to be sufficiently general to reflect the key features of sentience as they are currently understood and, with greater difficulty, to anticipate changes that may be required as the underlying scientific knowledge evolves.

The present analysis has highlighted currently understood key features of sentience-associated body functions and behaviour in animal taxa of welfare interest. ‘Welfare-aligned sentience’ is a characteristic of nervous systems that exhibit various levels of complexity. Yet, these nervous systems can all process sensory inputs in ways which generate a range of sensations, feelings, emotions or other subjective experiences (affects), and they also have a capacity to express and/or sustain a state or states of consciousness. These affects are experienced consciously and they manifest subjectively as either negative or positive. They reflect functional states within the body and/or the external circumstances of the animal, and they may motivate or otherwise be associated with the expression of life-sustaining behaviours. In this respect, subjective experiences ‘matter’ to the animal; but they also ‘matter’ because their ‘unpleasantness’ (negative) or ‘pleasantness’ (positive) has direct experiential impacts on the animal’s welfare. Another feature of welfare-aligned sentience which ‘matters’ to the animal is the subjective experiences that are derived from sensory inputs of touch, temperature, taste, smell, hearing, sight and/or other sensory modalities. These are integral to the animal’s capacities to communicate and interact with its own or different species, and to interact with its environment.

In vertebrates, the developing nervous system initially does not have a capacity to express sentience. During the subsequent intermediate developmental phase, it is unclear if brain function can support basic forms of sentience. However, once key functions of the mammalian cerebral cortex and equivalent avian structures become engaged, enhanced forms of sentience, as judged by the behaviour of the young, begin to be expressed. This occurs in different species several months, days-to-weeks, or minutes-to-hours after birth or hatching, these times being when the young need to respond behaviourally to unpredictable environments to which they are then exposed. At that stage, sentience-associated cognitive capacities underpin an increasing expression of volitional behaviours, the flexibility of which helps to secure the survival of the offspring at that time, and for the rest of its life. Some invertebrates (e.g., cephalopods, decapods) provide fairly strong evidence of welfare-aligned sentience, others (e.g., slugs, snails, bees, flies and ants) limited evidence of it; this has led the welfare of the former taxa to be acknowledged as meriting attention, but, as yet, not the latter taxa.

Accordingly, a succinct definition may now be offered for consideration: ‘welfare-aligned sentience’ confers a capacity to consciously perceive negative and/or positive sensations, feelings, emotions or other subjective experiences which matter to the animal. However, the utility of this definition with regard to the potential subtleties and/or limitations of its policy and legal implications remains to be determined. 

## 4. Deciding which Animals are Sentient should Focus on Both Negative and Positive Experiences

During the last 30 years, the welfare significance of sentience has been conceptualised mainly in terms of animals’ capacity to have negative affective experiences, especially pain and any associated suffering, with little attention given to positive experiences. This asymmetry may simply have reflected scientists’ preoccupation with welfare problem-solving conceived in terms of freeing animals from negative experiences and the internal states or external circumstances that elicit them [11,94,112]. It may also have arisen from an understanding that natural selection might have shaped emotions more for survival than for prosperity, there being many more ‘threats’ than ‘treats’ in the environment [134]. Indeed, pain and other putative aversive stimuli have been major foci of affect-centred research designed to explore the question of sentience in less-studied taxa including fishes [118,119,135], as well as cephalopods [56,128,129,130], decapods [136,137,138,139,140] and other invertebrates [50].

Based on increasing evidence generated during the last 10–15 years, sentient animals, principally mammals and birds, are now widely regarded as possessing a capacity to consciously experience both negative and positive affects (e.g., [7,9,11,37,40,45,49,94,99,101,107,125]). Thus, continuing to explore which animals are sentient, whether welfare-aligned or not, by mainly utilizing aversive stimuli should now be extended to devising ways to determine if the target animals can also have positive affective experiences [57]. The Five Domains Model [45,95] and the Five Provisions/Welfare Aims paradigm [94], as well as other approaches (e.g., [101]), may provide guidance on how this could be achieved. This wider understanding is beginning to receive some governmental recognition, for example, in commentaries on animal sentience in New Zealand [52,141] and Australia [142], but, as yet, not in law.

## 5. A Final Comment

Carefully evaluating sentience is crucial to our thinking about animal welfare, as welfare requires sentience. Accordingly, making well-informed decisions about which animals deserve our consideration and how that can be achieved requires an understanding of the key attributes of sentience, many of which have been outlined here.
